# Functional Magnetic Resonance Imaging Studies in Antisocial Personality Disorder: A Narrative Literature Review

**DOI:** 10.7759/cureus.63301

**Published:** 2024-06-27

**Authors:** Sharon Reyes, Sophie O'Hare, Elizabeth Lee, Rebekah Brown, Ievgen Chebotarov

**Affiliations:** 1 Department of Neuroscience, College of Medicine, Saint James School of Medicine, The Quarter, AIA

**Keywords:** brain scans, spect and pet imaging, fmri, psychopathy, cluster b aspd, antisocial personality disorder, cingulate gyrus

## Abstract

Individuals with antisocial personality disorder (ASPD) are characterized by impulsive behavior and a lack of empathy, which can eventually lead to criminal behavior. In our narrative review, we are investigating the neurological differences in the function, structure, and connectivity of those with ASPD in comparison to those without, with a major focus on psychopathic cluster B populations of ASPD individuals. We reviewed 86 published articles, and five of these met the inclusion criteria for distinct psychopathic and non-psychopathic groups. After reviewing these sources, we found deficits in function and structure leading to a lack of empathy and adherence to social norms in individuals with ASPD. Currently, there are very few treatment options for those living with ASPD. It is our opinion that with a better understanding of the structural and functional differences in those with ASPD, we might see more efficacious treatment therapies for this population group in the medical community.

## Introduction and background

Antisocial personality disorder (ASPD) is characterized by individuals who display manipulative, impulsive, and often criminal behavior. This disorder typically develops in early adolescence, emitting conclusive behavior patterns by the age of 15 and fully manifesting in the individual during their late 20s. A significant display of egocentric behaviors and lack of interpersonal skills are common characteristics of the disorder, along with a history of criminal activity. Pathological personality traits include deceit, manipulation, hostility, and an unpredictable nature. In addition to the behavioral traits found in those with ASPD, individuals with psychopathic traits and further characterization of ASPD exhibit emotional responses to fear and violence that are diminished [1]. They do not process fear and emotion in the same way healthy individuals do, further contributing to behaviors that non-psychopathic individuals would typically avoid for fear of consequences and repercussions. These behaviors are avoided in normal functioning brains due to what is known as averse conditioning.

Those considered in this review had a diagnosis of ASPD under the definition of the DSM-5TR (Diagnostic and Statistical Manual of Mental Disorder, Fifth Edition, Text Revision), which overlaps with both categories of psychopathy and sociopathy. While the DSM-5TR states that psychopathy and sociopathy are not equivalents of antisocial personality behavior, it allows for various convergences of psychopathy and sociopathy while focusing on antisocial personality traits [2].

According to the Diagnostic and Statistical Manual of Mental Disorders, Fifth edition, ASPD demonstrates a high heritability rate amongst first-degree relatives, along with environmental factors, including physical and sexual childhood abuse [3]. ASPD is a widespread issue across the United States, and individuals with this disorder have been known to make up to 80% of our correctional facility settings [4]. While in these correctional facilities, these individuals have been shown to have a poorer quality of life, as well as a higher rate of recidivism and suicidal risk [5]. Research has been completed regarding the profiling and conception of this disorder; the Natural History of Antisocial Personality Disorderprofiled ASPD as "a socially and irresponsible, exploitative, and guiltless behavior" [4]. Yet, a narrative review of how the brain functions within these individuals, while considering their positron emission tomography (PET)/single-photon emission computerized tomography (SPECT)/functional magnetic resonance imaging (fMRI) scans, has not been thoroughly researched and the data left uninterpreted. After reviewing this data in a broad sense, we believe that we will have a clearer picture of malfunctions or disruptions of the brain in individuals with ASPD and allow clinicians to better provide treatment plans for this patient population and potentially change the face of medicine.

To evaluate the role of the cingulate gyrus, a distinguished region of the prefrontal cortex in ASPD, data interpreting evidence in relation to psychopathy and the prefrontal cortex must be examined. In Koenigs' review of the role of the prefrontal cortex in psychopathy, the case of Phineas Gage was regarded as the first example of the effects on personality with damage to the prefrontal cortex [6]. Following the insertion of a metal rod through the frontal lobe, considering the inclusion of the prefrontal cortex, Phineas Gage's personality was said to have entirely changed. Following this, analysts were able to pinpoint a more specific area, the ventromedial prefrontal cortex, as a likely source of damage in individuals presenting with personality changes [6]. More specifically, changes in moral judgment, decision-making, irritability, and failure to learn from punishment were noted [6]. The ventromedial prefrontal cortex has been shown to mediate the connection of new possibilities with former experiences regarding reward and punishment. A decrease in the ability of the ventromedial prefrontal cortex to relate these connections in individuals with ASPD has been noted. A reduced volume of gray matter in the prefrontal cortex relates to the decreased ability to make neural synapses within these individuals [7]. These findings also relate to the personality changes discussed in Koenigs' review.

This review aims to focus on the role of the prefrontal cortex and limbic system in antisocial personalities. The cingulate gyrus, a portion of the limbic system, is thought to participate in the processes related to regulating behavior and emotions, specifically empathy, which is lacking in diagnosed individuals. We hypothesize that a broad review of research will show major differences between the brain of an individual diagnosed with ASPD to that of the general public due to many factors, including but not limited to differences in pathway and structure, neurotransmission, neural processing, and altered perception, as it relates intraneural connections between the prefrontal cortex and limbic system.

## Review

Search protocols

The search was conducted using the PubMed (Medline) database, as well as reviewing the following journals: New England Journal of Medicine, Nature, and Molecular Psychiatry Journal. The following MeSH search terms were used: [“Antisocial Personality Disorder” [Mesh] AND “Cingulate Gyrus” AND “fMRI” [MeSH] OR “SPECT” [MeSH]” OR “PET”]. The search period was 2000-2020. Only papers in English were chosen.

Criteria

There were a total of 83 articles screened for this review relating to Antisocial Personality Disorder. While reviewing these articles, we used the criteria described below for inclusion and exclusion parameters. Men and women aged 18-45, with a diagnosis of a psychotic disorder associated with ASPD were included in our review. We identified articles that utilized SPECT/PET/fMRI studies of individuals with antisocial personality disorder, focusing on articles with a clear non-psychotic and psychotic study group to compare imaging. When screening articles, we included all study designs in this review.

Information synthesis and search results management

**Figure 1:**
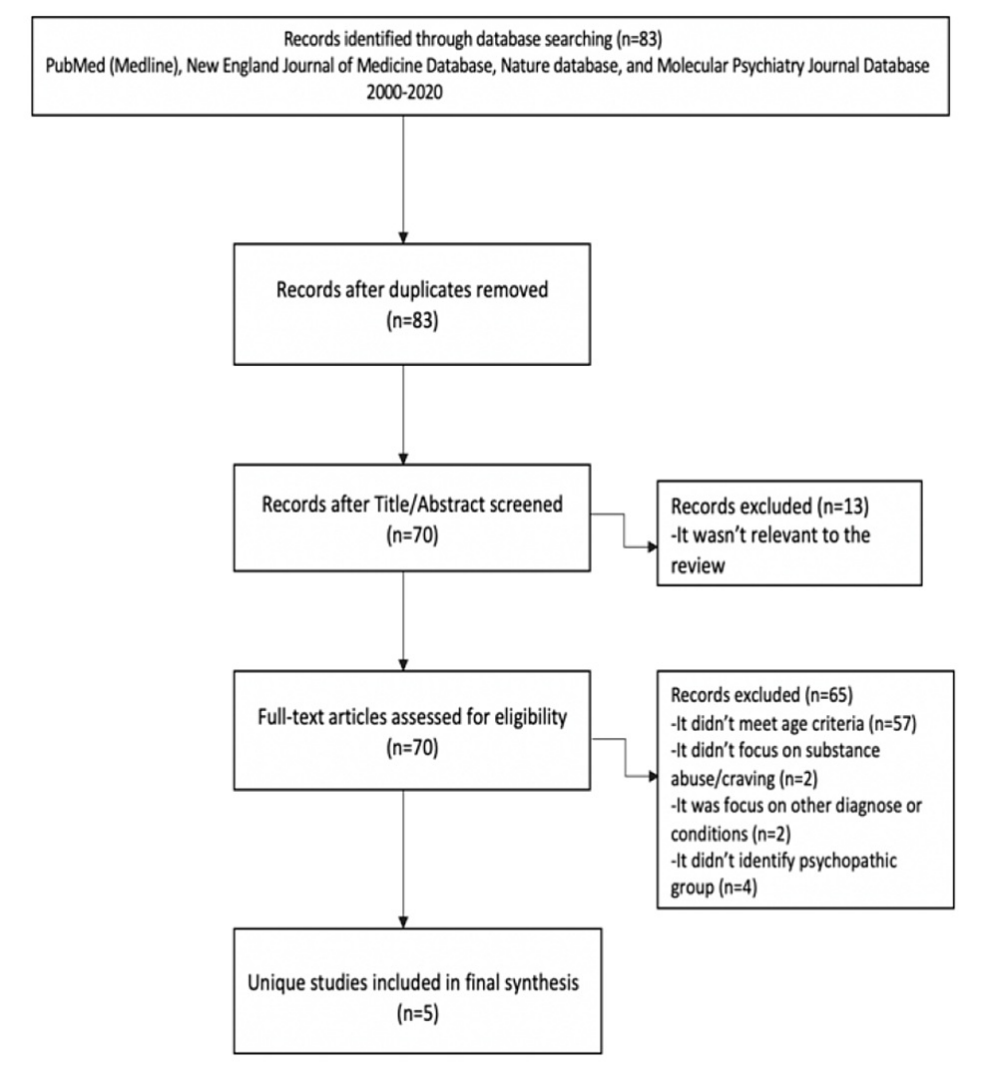
PRISMA flow chart PRISMA: Preferred Reporting Items for Systematic Reviews and Meta-Analyses

A manual review of each article was completed to evaluate if it fell within the outlined parameters and a summary of the results was created. We compared functional imaging data from diagnosed individuals with data obtained from healthy individuals. We noted the differences in the psychotic and non-psychotic testing groups in two main areas of interest, the prefrontal cortex and the limbic system. Differences that we recorded included alterations in brain activity, neurotransmitters, and cortical thickness. The Preferred Reporting Items for Systematic Reviews and Meta-Analyses (PRISMA) flow chart was used to track the stages of our process (Figure 1).

Because of the nature of our review, no experimental equipment or materials were necessary.

Results

Our search strategy yielded 83 papers. We excluded 13 articles because they were not relevant to our review. We then analyzed the remaining 70 papers for eligibility based on our inclusion criteria. We excluded 65 papers for the following reasons: 57 studies since they did not meet the age criteria, two studies that focused on substance abuse/craving, two studies that focused on other diagnoses or conditions, and four studies that were excluded due to a lack of an identified psychopathic group. Based on our inclusion criteria, this review finally comprised five papers dealing with functional neuroimaging in psychopathy, published from 2005 to 2017.

In our review, we included five articles, in which there were a total of 282 individuals that participated, broken down between 133 psychopathic individuals and 149 non-psychopathic individuals. All participants were males, many of them being inmates or on parole, between the ages of 18 and 45.

While we considered the following modalities: fMRI, SPECT, and PET scan, our articles were exclusive to fMRI. Four out of five of the studies we reviewed showed that there were some kind of functional or structural differences between the non-psychopathic and psychopathic populations (Table 1).

**Table 1:** Review of data fMRI: functional magnetic resonance imaging; OFC: orbital frontal cortex; CON: cortical network; ASPD: antisocial personality disorder

Name of the study, year	Imaging	Population		Prefrontal cortex (PFC) - dorsolateral, dorsomedial, ventrolateral, ventromedial, and orbitofrontal region	Limbic system - limbic cortex, cingulate gyrus, parahippocampal gyrus, hippocampal formation, the dentate gyrus, hippocampus, subicular complex, amygdala, septal area, hypothalamus
Cortical Thinning in Psychopathy, 2012 [8]	fMRI	Male Inmates, Ages 18-45	Non-psychopathic Group, N=31		- significant sustained activation of the frontolimbic circuit during leaning acquisition; -confirms fear conditioning involves this region
			Psychopathic Group, N=21	- lacks OFC activation when translating learned association into a behavioral response	-only right amygdala activation during learning acquisition -significantly reduced left amygdala activation during learning acquisition
Altered Resting - State Functional Connectivity in Cortical Networks in Psychopathy, 2015 [9]	fMRI	Male Inmates, Ages 18-45	Non-psychopathic Group, N=49		- there is a positive correlation with anxiety with regards to the CON connectivity and activity (specifically the anterior insula)
			Psychopathic Group, N=46		- suggests connectivity correlation between cortical associating hubs as a neurobiological marker of ASPD, - connectivity functionality dissociation between the dorsal anterior cingulate gyrus and the anterior insula, - normal connectivity within visual and auditory networks
No Volumetric Differences in the Anterior Cingulate of Psychopathic Individuals, 2016 [10]	fMRI	Males, ages 21-45	Non-psychopathic Group, N=24	- no significant volumetric differences were seen between the two groups	
			Psychopathic Group, N=24		
Deficient Fear Conditioning in Psychopathy: A Functional Magnetic Resonance Imaging Study, 2005 [11]	fMRI	Males, ages 23-41	Non-psychopathic Group, N=10		-there is enhanced activation in the limbic-prefrontal circuit at the acquisition of autonomic and verbal conditioning as well as the experience of fear
			Psychopathic Group, N=10		-no substantial activity to note; -no conditioned skin conductance and emotional valence ratings
Disrupted Functional Connectome in Antisocial Personality Disorder, 2017 [12]	fMRI	Males, ages 18-45	Non-psychopathic Group, N=335	- better integration of efficient information communication and integration of whole-brain networks	
			Psychopathic Group, N=32	- decreased functional connectivity within and between the left and right hemispheres; - increased path length and decreased network efficiency; - decreased modularity and different network modular organizations	

Discussion

When reviewing the fMRI results in the study “Cortical Thinning in Psychopathy” from 2012, it was evident that individuals with antisocial personalities had a significantly thinner cortex than non-psychopaths in several areas. The largest and most significant clusters were in the left insula, left dorsal anterior cingulate cortex, bilateral precentral gyrus, bilateral temporal pole, and right inferior frontal gyrus [8]. Thin cortical areas were not seen in the non-psychopathic group when compared to the psychopathic group. This study aimed to determine if there was any correlation between cortical thickness and psychopathy. MRI brain scans were taken of inmates to determine if there were structural differences in the cortical thickness between two groups, one group that had been diagnosed as psychopathic based on Psychopathy Checklist Revised (PCL-R) assessment results and the other group that was identified as non-psychopathic. It was determined that the psychopathic group had a thinner cortical area than the control group in up to 13 areas of the brain.

There also appeared to be decreased connectivity in pathways of the brain between the left insula and left dorsal anterior cingulate cortex. The results of this study seem to support two of the models currently at the forefront of research. One shows that psychopaths exhibit a brain dysfunction related to circuitry between the amygdala and the ventromedial prefrontal cortex. The other indicates that psychopaths exhibit brain dysfunction in the paralimbic network.

**Figure 2:**
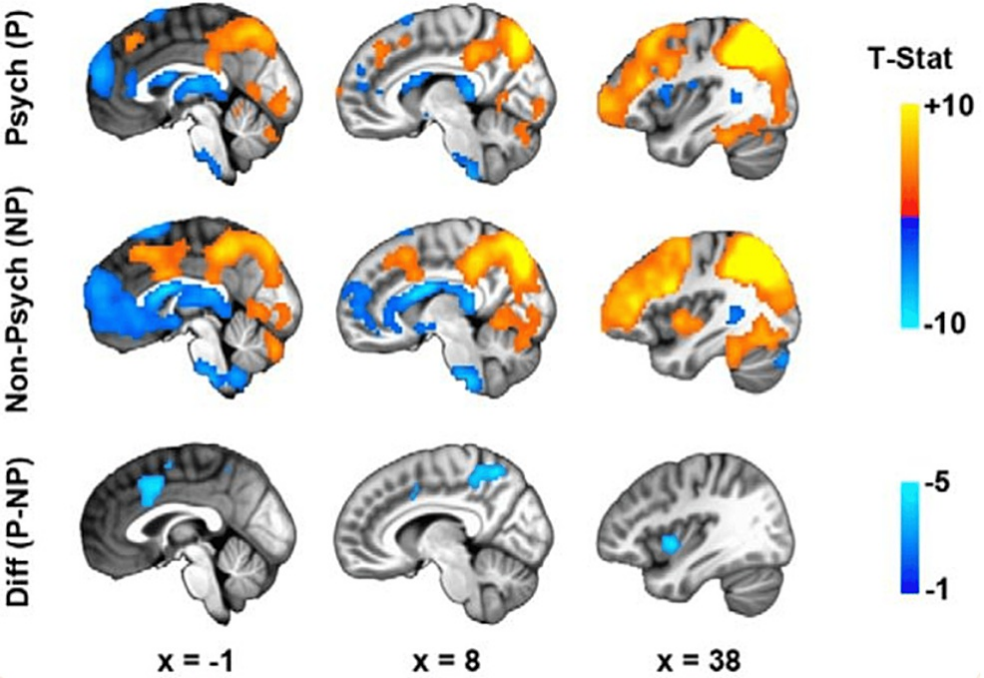
Functional connectivity differences in psychopaths vs non-psychopaths Source: [9] Published under Creative Commons Attribution 4.0 International License (CC-BY)

In the study “Altered Resting-State Functional Connectivity in Cortical Networks in Psychopathy,” a decreased connection is seen between the parietal and frontal regions and appears to be the principal factor in determining the severity of the psychopathy (Figure 2). This reduced network is a major cause of psychopathic traits [9]. Interpersonal/affective traits were linked exclusively to decreased cortical connectivity whereas lifestyle/antisocial traits were linked exclusively to increased cortical connectivity. The researchers suggest connectivity correlations between cortical associating hubs as a neurobiological marker of ASPD. It was noted that there seemed to be a dissociation in the normal functioning between the dorsal anterior cingulate cortex and the anterior insula in the psychopathic group. There did appear to be normal connectivity within visual and auditory networks in the psychopathic group compared to the non-psychopathic group.

In the 2010 article “No Volumetric Differences in the Anterior Cingulate of Psychopathic Individuals,” the authors suggest that functional imaging in those with psychopathy shows reduced anterior cingulate activities. Results appeared unclear as to whether the anterior cingulate would be considered structurally impaired [10]. There were no volumetric differences seen in the anterior cingulate, nor in its ventral and dorsal regions. This study was the only one within our research that did not find a significant difference in brain structure or connectivity between the non-psychopathic and psychopathic groups.

In Birbaumers' 2005 article, “Deficient Fear Conditioning in Psychopathy: A Functional Magnetic Resonance Imaging Study”, the authors indicate that psychopathic behavior might be the consequence of deficient fear conditioning. Results suggested that in the circuit between the prefrontal (anterior cingulate, insula, orbitofrontal cortex, amygdala) and limbic regions, there was significant activation seen in the healthy controls upon successful autonomic and verbal conditioning and during experiences of fear [11]. The difference in brain activity between the psychopathic and non-psychopathic groups was significant. There was minimal activity noted in the circuit imaging of the psychopathic group. Although the ratings of contingency, knowledge based on the highest outcome probability of an event, and arousal appeared to be normal, the psychopath group failed to show emotional valence ratings and did not exhibit conditioned skin conductance through the duration of the testing.

According to the study from 2017, “Disrupted Functional Connectome in Antisocial Personality Disorder,” a substantial neurological deficit in ASPD patients was seen when compared to a healthy control group [12]. Patients with ASPD had a significant reduction in the frontal-subcortical and frontoparietal modularity as well as a significantly reduced connectivity in these same regions. Disturbances were primarily observed between the gyri located in the frontal and parietal regions and also in the pathways between these two gyri and other regions of the brain. It is speculated that these disturbances in this control network, which lead to a decrease in the functional integration in the brain, could also account for deficits in cognition in patients with ASPD. In ASPD patients, network efficiency as well as an increased length in the path was revealed by the small-world analysis. The implication of this is that there is reduced integration for functions that use the whole brain. It can be theorized that the neurological disruptions experienced by ASPD patients are connected with the topological organization of functioning networks in the brain, specifically the frontoparietal network, and how they are segregated, as well as a reduction in the integration of the brain. This would play a role in the behavioral and cognitive disturbances that are seen in patients with ASPD.

Some of the significant findings in the prefrontal cortex area seen in non-psychopathic groups were sustained activation of the frontolimbic circuit during learning acquisition; confirmation of fear conditioning involving the prefrontal cortex; positive correlations between increased activity within the anterior insula and altered activity within the cingulate gyrus with anxiety [13]; and enhanced activation in the limbic-prefrontal circuit during acquisition of fear and verbal/autonomic conditioning.

In the psychopathic population reviewed, we saw: selective activation during learning acquisition specific to the right amygdala; significantly reduced left amygdala activation during learning acquisition; connectivity correlations between cortical associating hubs as a neurobiological marker of ASPD; disconnection between the dorsal anterior cingulate cortex and the anterior insula with regards to the functional connectivity; normal connectivity within visual and auditory networks; and no conditioned skin conductance and emotional valence ratings. These aspects are relevant as the prefrontal cortex is known for its part in cognitive control function and its influences on attention, impulse inhibition, memory, and cognitive flexibility. 

The limbic system is the part of the brain involved in our behavioral and emotional responses especially related to our survival. When reviewing our non-psychopathic group in relation to this system, we found better integration of efficient information communication and integration of whole-brain networks; and fluid orbital frontal cortex (OFC) activation when moving learned associations to a behavioral response. The psychopathic group revealed: a lack of OFC activation when translating learned associations into a behavioral response; decreased functional connectivity within and between the left and right hemispheres; increased path length and decreased network efficiency; and decreased modularity and different network modular organizations within this region of the brain.

Based on these studies, we believe it would be beneficial to investigate programs that identify some of these common neural abnormalities and traits earlier on to better facilitate a healthy support system for individuals suffering from ASPD and psychopathic behaviors. Areas that could use further research include a closer look at cortical thickness and the role of the anterior cingulate cortex in psychopathy and its correlations with ASPD. We think future research could expand upon this review by increasing the age range and looking at the differences in the brain as a whole, rather than isolating specific areas of interest.

## Conclusions

In conclusion, several differences were observed in fMRI when comparing the psychopath to the non-psychopath. These differences include, but are not limited to, cortical thinning, decreased brain connectivity between brain regions, most often between the regions of the frontal and parietal lobes and decreased activity in the circuit between the limbic and prefrontal regions. Analysis of the research indicates that these differences are useful in explaining many of the behavioral traits and cognitive deficits seen in those with ASPD.

## References

[1] Sommer M, Hajak G, Döhnel K, Schwerdtner J, Meinhardt J, Müller JL (2006). Integration of emotion and cognition in patients with psychopathy. Prog Brain Res.

[2] Conti RP (2016). Psychopathy, Sociopathy, and Antisocial Personality Disorder. Forensic Res Criminol Int J.

[3] American Psychiatric Association (2013). Diagnostic and Statistical Manual of Mental Disorders, 5th ed.. Diagnostic and Statistical Manual of Mental Disorders, 5th ed.

[4] Black DW (2015). The natural history of antisocial personality disorder. Can J Psychiatry.

[5] Black DW, Gunter T, Loveless P, Allen J, Sieleni B (2010). Antisocial personality disorder in incarcerated offenders: psychiatric comorbidity and quality of life. Ann Clin Psychiatry.

[6] Koenigs M (2012). The role of prefrontal cortex in psychopathy. Rev Neurosci.

[7] Raine A, Lencz T, Bihrle S, LaCasse L, Colletti P (2000). Reduced prefrontal gray matter volume and reduced autonomic activity in antisocial personality disorder. Arch Gen Psychiatry.

[8] Ly M, Motzkin JC, Philippi CL, Kirk GR, Newman JP, Kiehl KA, Koenigs M (2012). Cortical thinning in psychopathy. Am J Psychiatry.

[9] Philippi CL, Pujara MS, Motzkin JC, Newman J, Kiehl KA, Koenigs M (2015). Altered resting-state functional connectivity in cortical networks in psychopathy. J Neurosci.

[10] Glenn AL, Yang Y, Raine A, Colletti P (2010). No volumetric differences in the anterior cingulate of psychopathic individuals. Psychiatry Res.

[11] Birbaumer N, Veit R, Lotze M, Erb M, Hermann C, Grodd W, Flor H (2005). Deficient fear conditioning in psychopathy: a functional magnetic resonance imaging study. Arch Gen Psychiatry.

[12] Jiang W, Shi F, Liao J (2017). Disrupted functional connectome in antisocial personality disorder. Brain Imaging Behav.

[13] Alvarez RP, Kirlic N, Misaki M, Bodurka J, Rhudy JL, Paulus MP, Drevets WC (2015). Increased anterior insula activity in anxious individuals is linked to diminished perceived control. Transl Psychiatry.

